# 

**Published:** 1903-10

**Authors:** 


October, 1903. VOL. XXIV. No. 10.

### THE INTERNATIONAL DENTAL JOURNAL.

LIBRARY
Indexed
S.G.O.

JAMES TRUMAN, D.D.S., EDITOR,
PHILADELPHIA.

*COLLABORATORS:*

WILLIAM H. POTTER, A.B., D.M.D., BOSTON.
CHARLES P. BRIGGS, A.B., M.D., D.M.D., BOSTON.

LIBRARY
SURGEON GENERAL'S OFFICE
-3-OCT.-1903

### Summary of Contents.

(*For details, see p. 2.*)

ORIGINAL COMMUNICATIONS.
DOMESTIC CORRESPONDENCE.
FOREIGN CORRESPONDENCE.
EDITORIAL.
MISCELLANY.
BIBLIOGRAPHY.
OBITUARY.
CURRENT NEWS.
REPORTS OF SOCIETY MEETINGS.

*$2.50 a Year, in Advance. Single Copies, 25 Cents.*

PUBLISHED MONTHLY BY

The International Dental Publication Company,

227-231 SOUTH SIXTH ST., PHILADELPHIA.

EDITORIAL OFFICE, 4505 CHESTER AVENUE, PHILADELPHIA, PA.

PRINTED BY J. B. LIPPINCOTT COMPANY, PHILADELPHIA.

Entered at the Post-Office at Philadelphia, and admitted for transmission through the mails at second-class rates.

